# Papillary thyroid microcarcinoma (Black Ink)

**DOI:** 10.18632/oncotarget.25621

**Published:** 2018-06-26

**Authors:** Ersilio Trapanese, Carmine De Bartolomeis, Basilio Angrisani, Giulio Tarro

**Affiliations:** ^1^ Interventional Ultrasound of Breast Oncology Screening, ASL Salerno, Salerno, Italy; ^2^ Endocrine Surgery and General Surgery Specialist, University Pisa, Pisa, Italy; ^3^ Anatomical Pathology Specialist, University Hospital of Campania “L. Vanvitelli”, Naples, Italy; ^4^ President Foundation T. & L. de Beaumont Bonelli for Cancer Research, Naples, Italy; ^5^ Chairman of the VirusSphere World Academy of Biomedical Technologies (WABT) UNESCO, Paris, France

**Keywords:** ultrasonography, ADF, FNAC, thyroid microcarcinoma, Black Ink

## Abstract

We report a case of a 58-year-old Caucasian woman affected by papillary thyroid microcarcinoma (PTMC) of the left-lobe of the gland with very small size (Ø 0.3 cm). The characteristics with the Diagnostic Imaging using Ultrasonography, ADF (Advanced Dynamic Flow), and fine-needle-aspiration cytology (FNAC) are discussed, comprising a very small micro-focus of radial shape, with markedly hypoechoic echostructure, irregular margins, supplemented by peripheral vessel formation. It acquires an image which appears similar to a brisk visualization of a dark ink stain in the normal thyroid weave. We call such a pattern “Black Ink” with ultrasonographic image and believe consistent with the infiltrating variant of papillary thyroid microcarcinoma if associated with malignant cytology after FNA.

## INTRODUCTION

According to WHO Papillary Microcarcinoma is any thyroid papillary carcinoma with a diameter of 1 cm or less [1]. These tumors are occasionally diagnosed as an incidental finding after thyroidectomy for other pathology or autopsy; some of these very small cancers are completely encapsulated and tend to behave indolently while some others clearly infiltrate the thyroid parenchyma. Malignant behavior has been proven by several authors [2, 3, 4].

Clinical-pathological features and prognosis of this neoplasm, have been discussed in a previous study [5].

The most affected topographic anatomical area is the middle third of the right or left thyroid lobe. The tumor has mainly a woman distribution, with the age range between 27 - 75 years [6, 7]. The cause of papillary carcinoma is unknown, there are risk factors for the development of thyroid cancer (ionizing radiation, iodine deficiency, autoimmunity, familiarity) [8, 9].

Ultrasonography represents the diagnostic technique for more sensitive images for an early diagnosis of thyroid lesion [10, 11, 12, 13].

The ADF (Advanced Dynamic Flow) allows to underline the vessel flow of the newly formed tortuous vessels [14].

The FNAC (Fine Needle Aspiration Cytology) has allowed the diagnosis with a 92-95% diagnostic accuracy (positive predictive value based mainly on the nuclear features of the cancer cells) [15].

We present a case of a infiltrating papillary thyroid microcarcinoma, size 0.3 cm, identified on the ultrasound examination and ADF in the left of the thyroid lobe.

## CASE REPORT

A 58-year-old Caucasian woman with no personal history of thyroid disease presented herself to perform a thyroid ultrasound examination.

Routine laboratory tests results were normal. BMI result was 33 kg/m2.

A sister was previously diagnosed of papillary thyroid carcinoma.

Bidimensional ultrasonography (DUS 2) using high frequency probes (13 - 15 MHz) (Toshiba Aplio 500) highlighted a highly suspicious micro-focus of the left lobe, of radial shape with markedly hypoechoic echostructure, irregular margins, size 0.3 cm (classified TI-RADS 5, very high risk lesion ATA guidelines).

(Figure 1-5).

**Figure 1:**
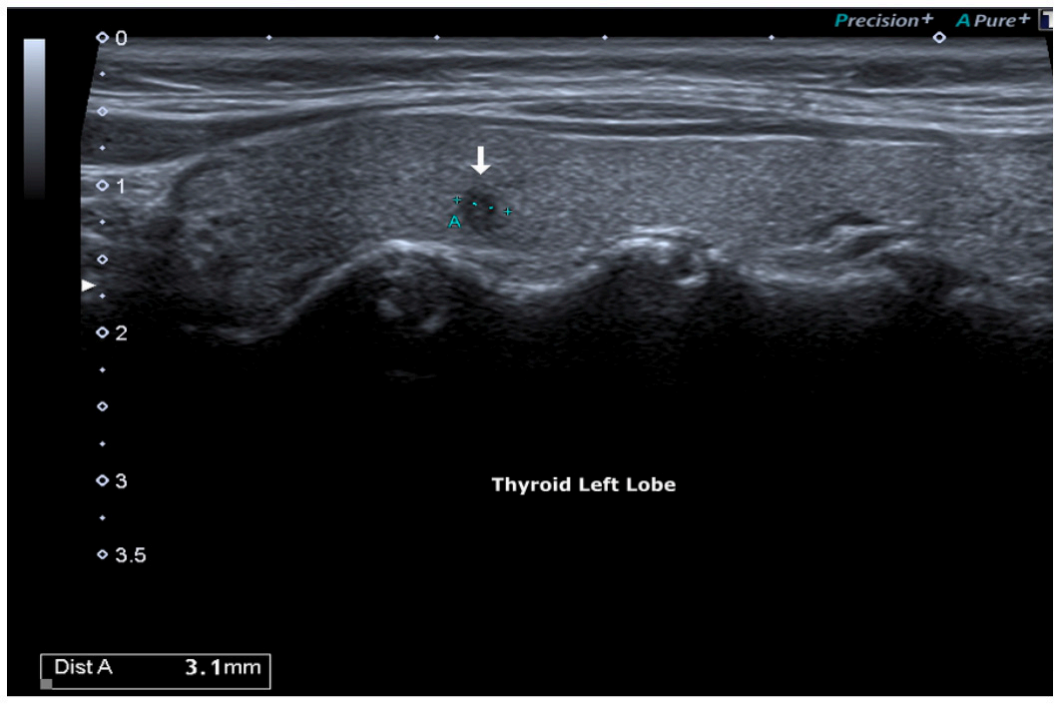
Hypoecogenic micro-focus, irregular margins

**Figure 2:**
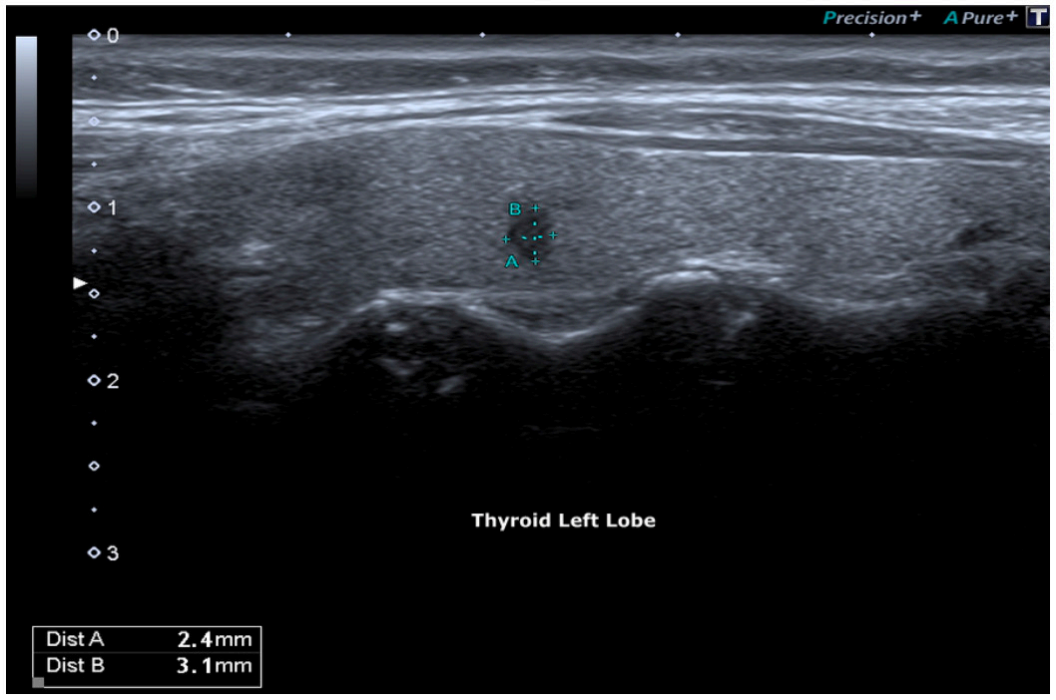
Micro-focus, irregular margins: 3 mm

**Figure 3:**
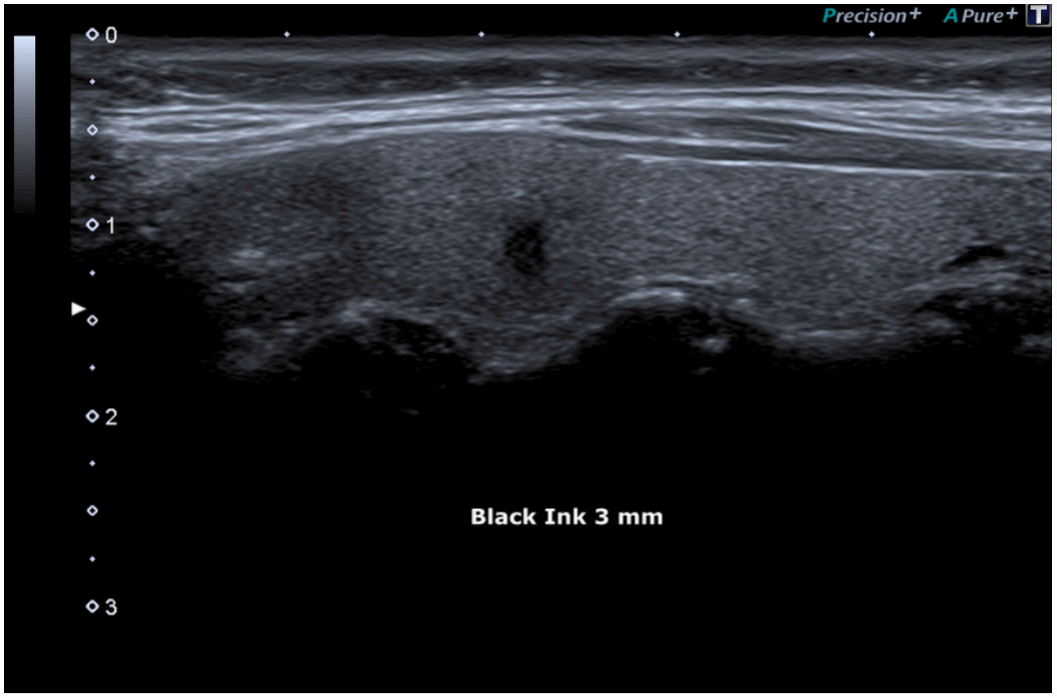
Black Ink

**Figure 4:**
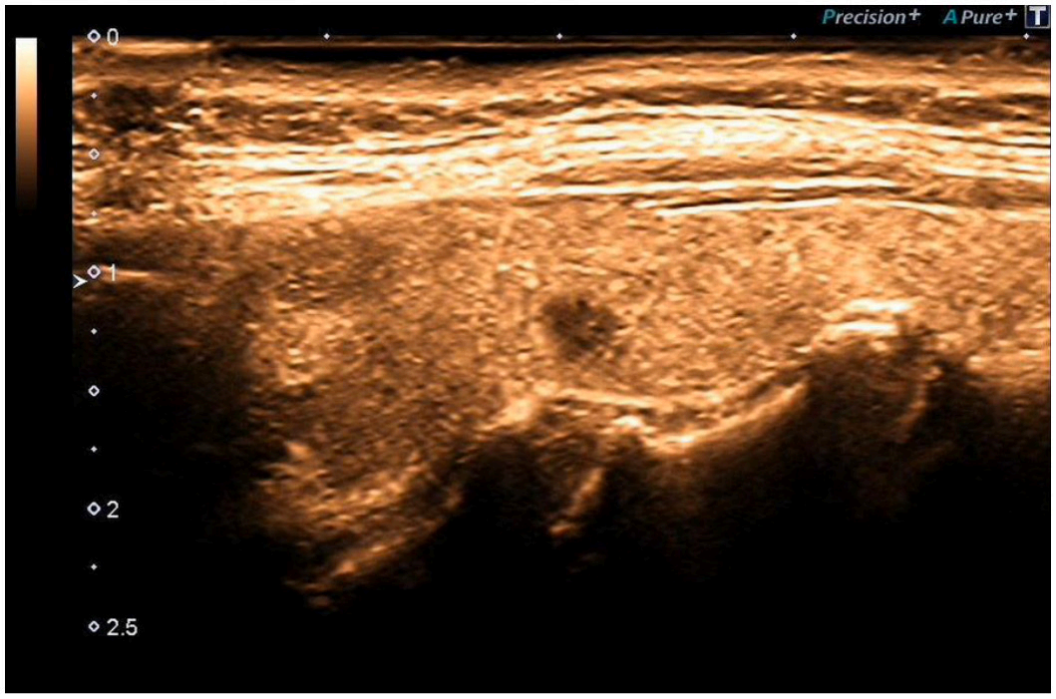
Black Ink

**Figure 5:**
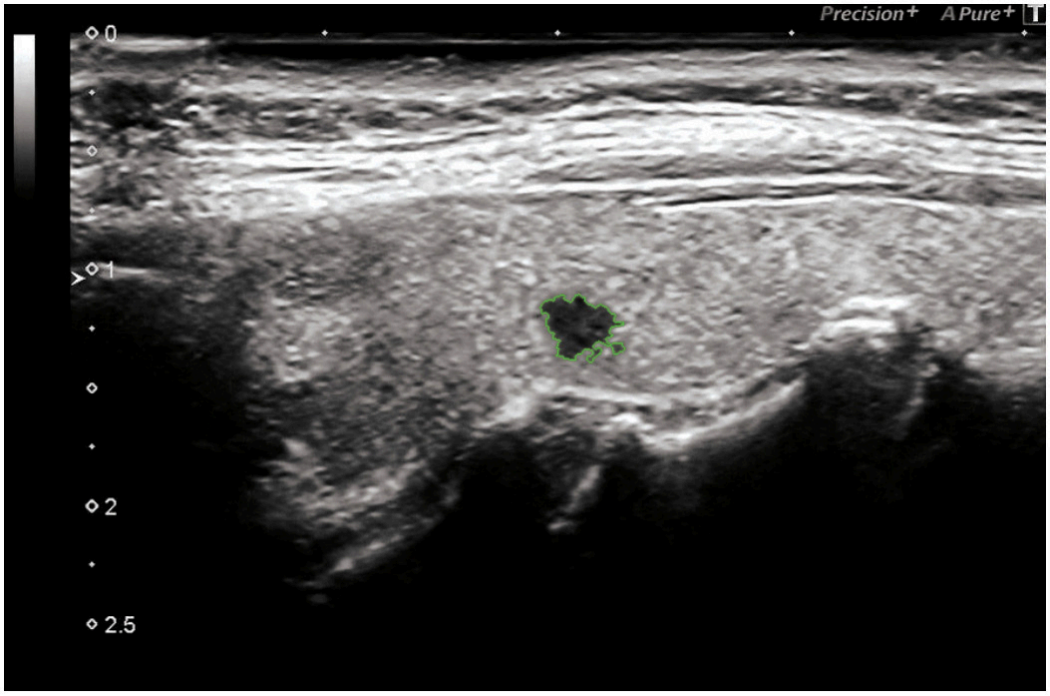
Black Ink, irregular margins: 3 mm

**Figure 6:**
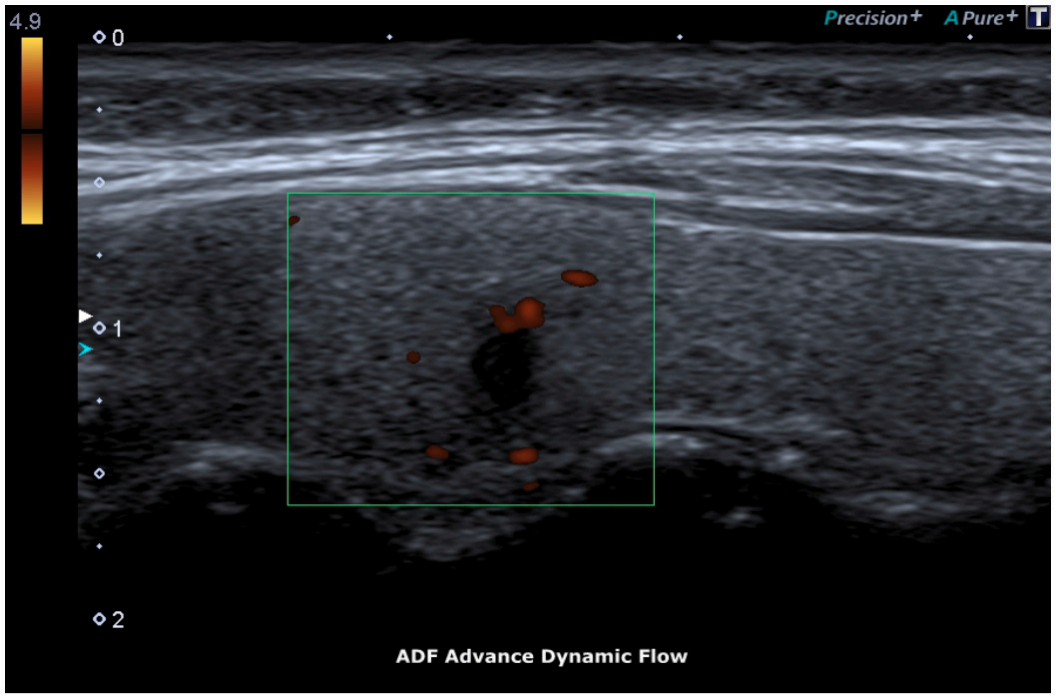
ADF, Advance Dynamic Flow

**Figure 7:**
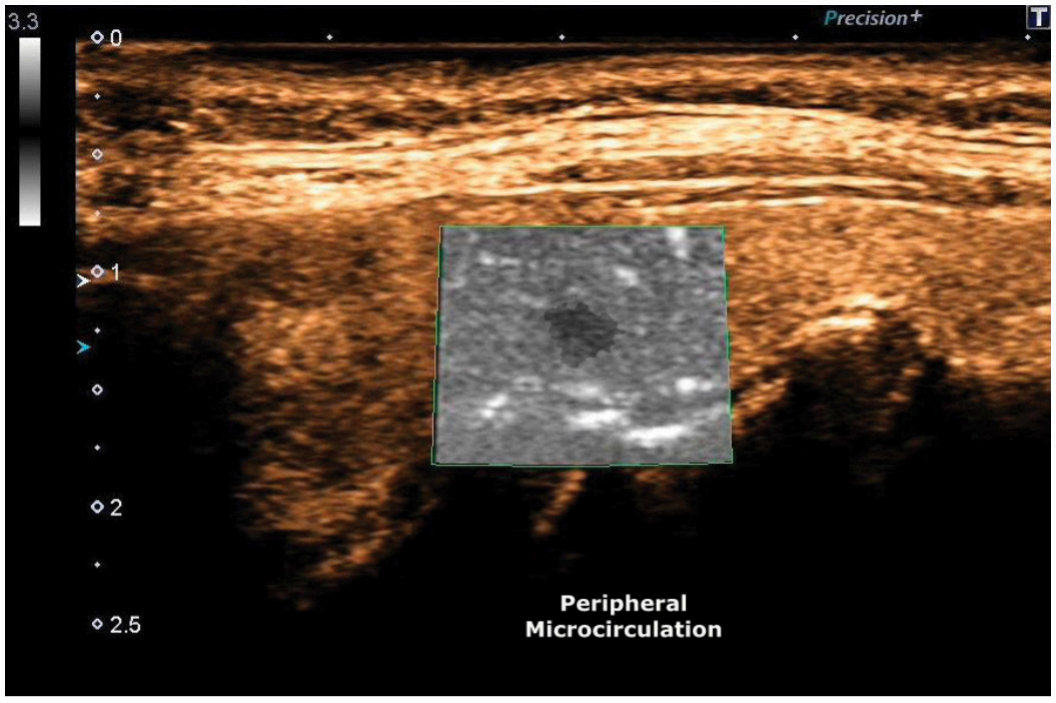
ADF, Peripheral Microcirculation

ADF test showed a clear flow through newly formed tortuous vessels at the periphery of the specific micro-focus (Figure 6, 7).

**Figure 8:**
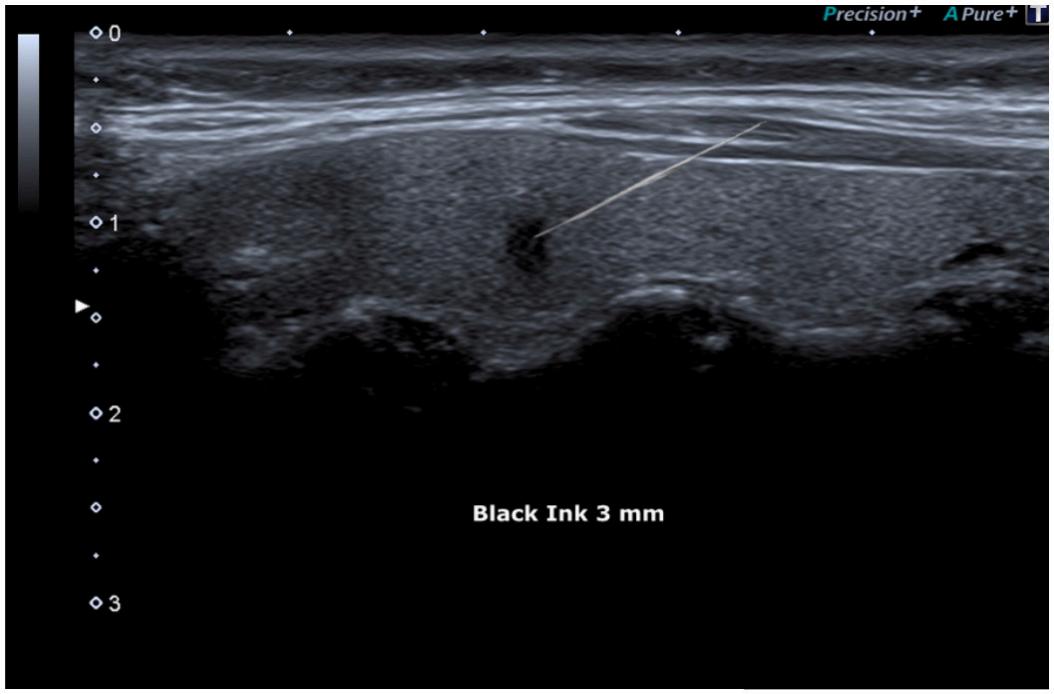
FNAC (Fine Needle Aspiration Cytology) Showed under guide

FNAC procedure was proposed to the patient and, after informed consent had been obtained, it was performed under ultrasound guidance (Figure 8).

**Figure 9:**
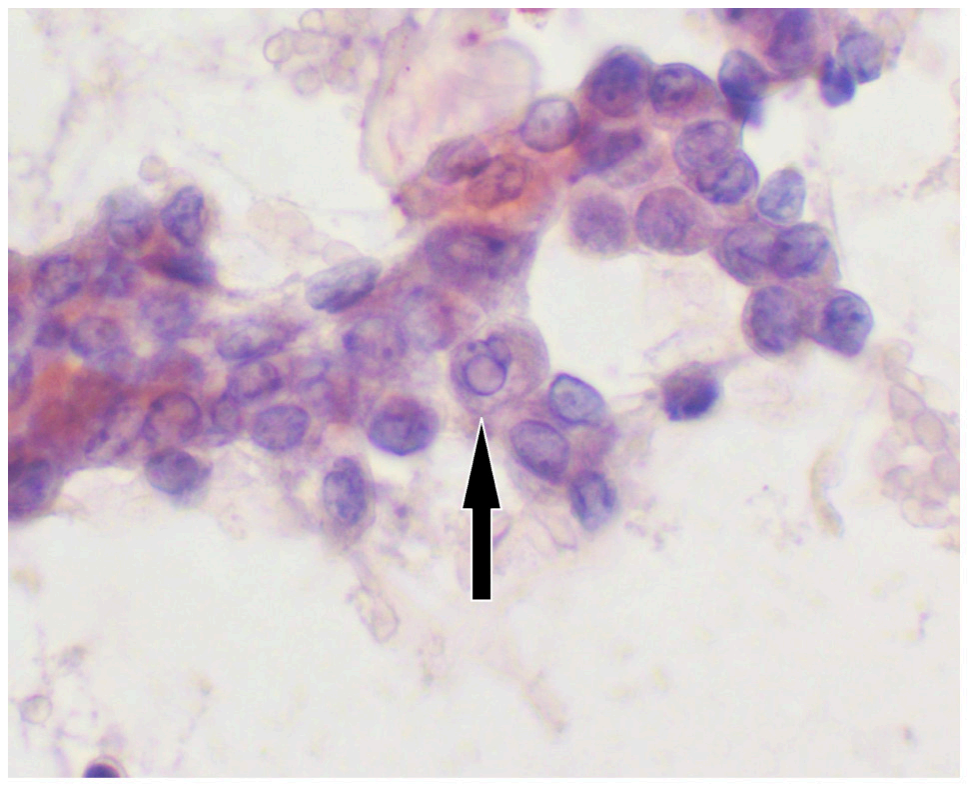
Zoom Cytology image This illustrative cell group shows most important features of papillary carcinoma:nuclear grooves and pseudoinclusion.

**Figure 10:**
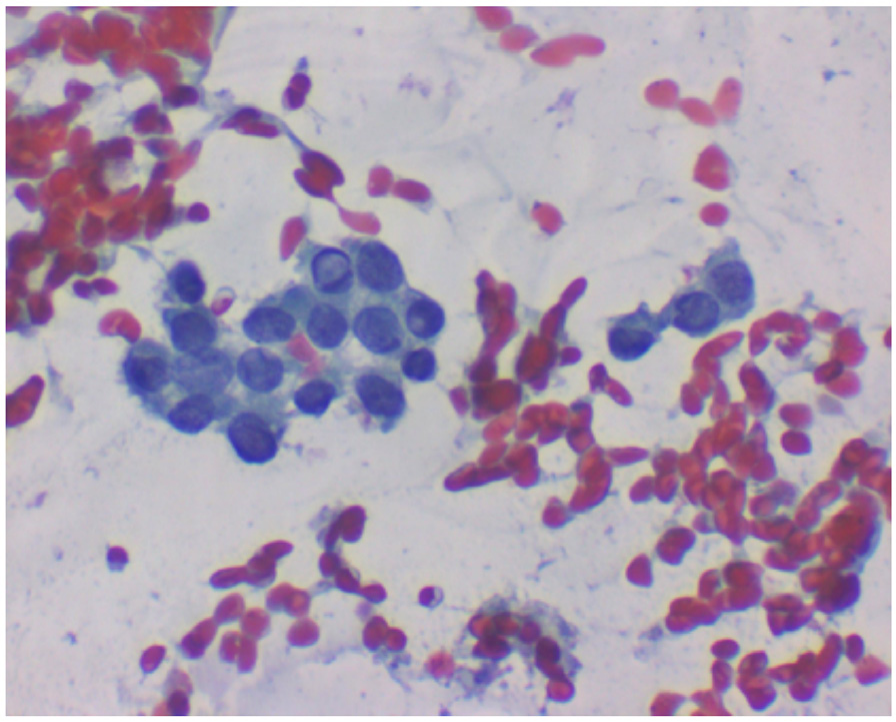
(600x) It shows clusters of cohesive epithelial cells with enlarged nuclei, incremented nuclear/cytoplasmicratio, nuclear crowding and presence of prominent nuclear grooves and pseudoinclusions

Cytologic examination of the slides (Papanicolaou stain) allowed recognition of malignancy with cytologic pattern very suspicious for papillary tumor (TIR5 cathegory according to Italian Consensus, cathegory VI according to The Bethesda System for Reporting Thyroid Cytopathology (Figure 9, 10).

The patient became candidate for thyroidectomy and subsequently underwent a Total Extracapsular Thiroidectomy procedure (TT). Some enlarged lymph nodes (ENL) were evaluated ultrasonography previous to surgical exploration, and found and removed close to the left inferior thyroid pole at the time of thyroidectomy.

Histological examination of the lesion showed a proliferation of well differentiated epithelial cells forming an purely papillary structure surrounded by blood vessels, with irregular borders, overtly infiltranting thyroid parenchyma.

**Figure 11:**
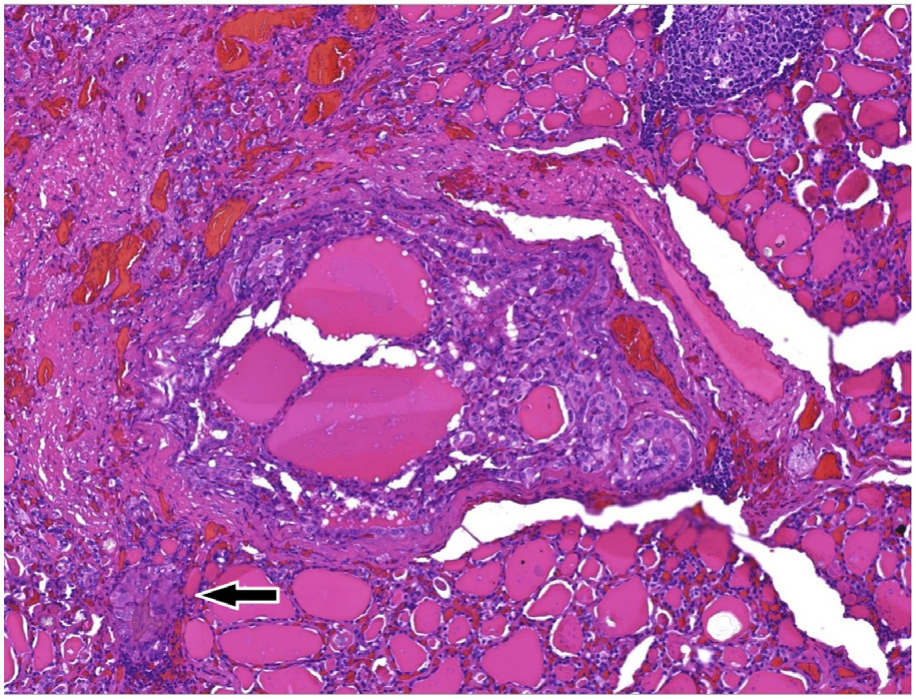
(100x) Whole section of the PTMC, classic variant, Ø 0.3 cm, devoid of a capsule structure, being made offollicoles and well formed papillae with fibrovascular core, irregular contour, surrounded by newly formed microvessels White arrow highliths a papillary group of neoplastic cells penetrating normal thyroid follicoles at the periphery of the main focus, which indicates that the tumor already shows tendency to spread in the surrounding (invasion).

The neoplasm showed no relationship to thyroid capsule and no vascular or lymphatic invasion. All the lymph nodes retrieved were devoid of metastatic cells (final TNM 8^th^ eds. staging pT1, pN0) (Figure 11).

Patient underwent hormonal and tumor markers evaluation plus ultrasonographic evaluation of the neck after 3 months. Post operative remnants therapy with Radioiodine I-131 (RAI), was not administered inasmuch the tumor was very little, contained in the thyroid and lacked vascular invasion.

## DISCUSSION

Zhao [16] and Ghossein [17] argue that the difference of the papillary microcarcinoma is only for the size in comparison with other thyroid cancers and not for morphological, clinical and prognostic characteristics.

They report several cases of microcarcinoma followed by recurrence and aggressive behaviour with multiple metastases.

Kasai and Sakamoto [18] claim that the sizes of PTMC are markers of limph node metastasis and vascular invasion.

The micro-focus identified was localized at the middle third of the left lobe, presenting a radial shape, markedly hypoechoic echostructure, irregular margins, size 0.3 cm, characteristics with a high predictive for malignancy value (p <0.001) [19].

The ADF allowed detection of microvascularization at the periphery of the micro-focus with accuracy.

Despite the small size of the lesion, the diagnostic image was strongly suspicious for malignancy and the FNAC procedure determined accurate detection of malignancy. Based on nuclear crowding and presence of prominent nucleargroves and pseudoinclusions FNAC was classified TIR5 according to the Italian consensus [20] and Category VI according to the Bethesda Classification [21].

Age and gender are considered prognostic factors for the recurrence and survival of PTMC patients [22, 23, 24].

In papillary thyroid microcarcinoma (PTMC), a higher recurrence rate was noted in patients with a familiar history of PTMC, this remained predictive and indicates an increase in biological aggressiveness [25].

According to Hay [26] two are the important parameters to keep in mind in the possible onset of a reccurence: multifocality (number of focus) and the type of surgical treatment ( total thyroidectomy versus lobectomy).

Nevertheless, some authors suggest that there exist a subset of PTMCs that can be aggressive, requiring therapeutic management similar to larger tumors ( PTCs) [27, 28].

Familiarity, age and gender, for patients with these characteristics of high risk of long-term recurrence, total extracapsular thyroidectomy (TT), following the technique described by Lahey [29] and dissection of some enlarged lymph nodes (ELN), close to the left inferior thyroid pole, was considered to avoid reoperation (Hwangbo et al., 2017) [30]. (Supplementary Figures 1, 2).

Yokozoa [31] and Ahuia [32] have respectively documented that 15,9% of cancers less than 1 centimetre show an extra thyroid invasiveness and that occult metastasis of thyroid cancer to the limph nodes is up 20% of cases.

Cut surfaces showed a whitish equatorial nodule of 0.3x0.2x0.2 cm in the left lobe.

On histological examination the lesion was classified as papillary thyroid microcarcinoma (PTMC, classic variant), the neoplasm beeing made of well formed papillae intermingled focally with normal thyroid parenchima. Lymph node showed no involvement by tumor cells, and pathological stage was pT1a, pN0 according to TNM 8th edn [33, 34, 35].

The tumor characteristics of the “Black Ink”, highlighted in this case report, provides a reliable prognostic classification, in order to define an effective therapeutic plan.

Despite the small size of this micro-focus, dimension 0.3 cm, this lesion is a variant of the papillary thyroid cancer with a potential aggressive behavior [36, 37, 38].

Being by nature, an infiltrating type, it is commonly classified in the dimensional group of small papillary tumors [39].

For these reasons, this case report has implemented an accurate analysis of the histological characteristics and of the long-term prognosis, underlining the differential diagnosis between the differentiated papillary microcarcinoma and the microcarcinoma infiltrating type. Mixing these different pathological entities within a common definition of “Papillary Thyroid Microcarcinoma (PTMC)” creates confusion in the communication process, and potentially compromises the management of a well-measured therapy in individual cases.

In recent years, several clinical and histologic risk factors for aggressiveness have been identified in PTMCs, such as size ≤0.5 cm, multifocality, tumor extension beyond the parenchyma, limph node involvement [40].

Hawk [41] argues that size appears to be an important determinant of biological behavior of papillary carcinomas of the thyroid.

The optimal values of the size still remain controversial.

Lim et al., (2009) [42] indicated it would be 0.7 cm, Zhang et al., (2012) [43] indicated it would be 0.6 cm, and Chang et al. (2015) [44] indicated it would be 0.5cm.

It is very important that the size must be cautiously interpreted in the current increasing subgroup of PTMCs to verify which diameter would be more representative of the malignancy risk of nodules.

Wang et al. claim that the size of PTMC in ultrasound images are helpful to predict the aggressiveness of the tumors, it may become an easy predictor for PTMC prognosis and assist us to choose treatment (Wang et al., 2015) [45].

## CONCLUSIONS

Thyroid cancer is among the most common cancers in women. We described a case of a 58-year-old woman with a Ø 0.3 cm “**Black Ink” image,** which was subjected to FNAC and cytology examination and treated with total thyroidectomy with subsequent histology report of papillary thyroid microcarcinoma (invasive type). Ultrasonography together with FNAC have proven to be the most sensitive diagnostic imaging techniques for the early diagnosis of this thyroid neoplasm.

We are convinced that, despite the very small size of this micro-lesion, Black Ink echopattern represents a very important biological risk factors and that size, actually, has a serious consideration in malignancy assessment.

Diagnostic Imaging is a highest tool for early diagnosis in order to change the course of the disease. We point out the important acquisition of a well structured informed consent prior to surgery, which prospects a small chance that before thorough sectioning of the surgical specimen, such small lesions could be missed or be technically not feasible by pathology techniques.

## SUPPLEMENTARY MATERIALS FIGURES


