# Supplementary material for: Papillary thyroid microcarcinoma (Black Ink)
Source: Oncotarget. 2018 Jun 26;9(49):29275–83. doi: 10.18632/oncotarget.25621 (PMC6044379; doi:10.18632/oncotarget.25621)
Supplement: Supplementary file 1 [file oncotarget-09-29275-s001.pdf]

Papillary thyroid microcarcinoma (Black Ink)

SUPPLEMENTARY MATERIALS

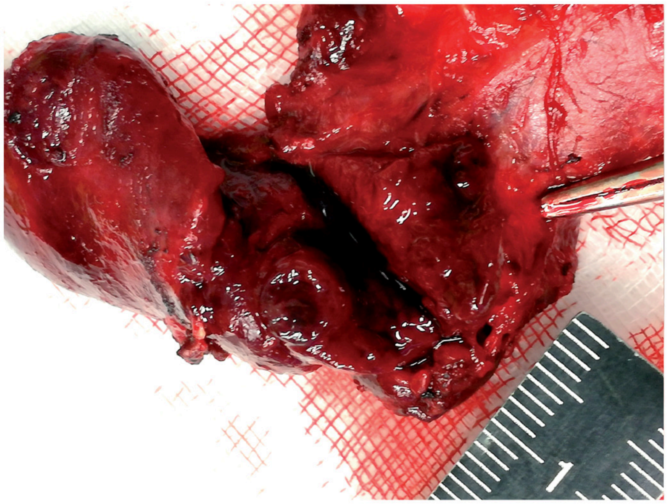

Supplementary Figure 1: Macroscopic Pathology.

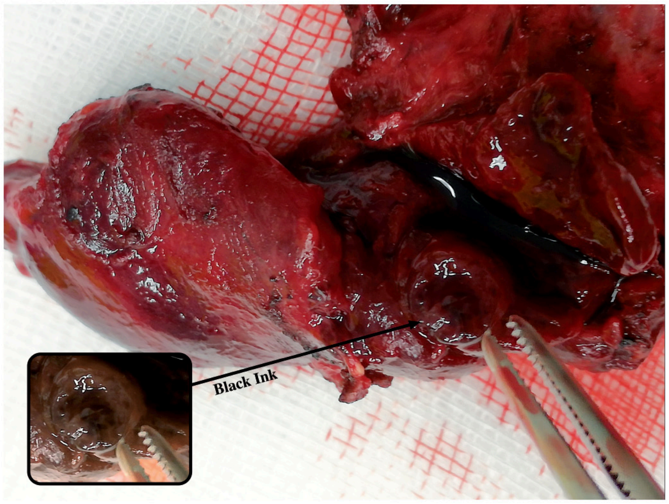

Supplementary Figure 2: Zoom Black Ink image: Position Area Left Lobe.
